# HIV epidemiologic trends among occupational groups in Rakai, Uganda: A population-based longitudinal study, 1999–2016

**DOI:** 10.1371/journal.pgph.0002891

**Published:** 2024-02-20

**Authors:** Victor O. Popoola, Joseph Kagaayi, Joseph Ssekasanvu, Robert Ssekubugu, Grace Kigozi, Anthony Ndyanabo, Fred Nalugoda, Larry W. Chang, Tom Lutalo, Aaron A. R. Tobian, Donna Kabatesi, Stella Alamo, Lisa A. Mills, Godfrey Kigozi, Maria J. Wawer, John Santelli, Ronald H. Gray, Steven J. Reynolds, David Serwadda, Justin Lessler, M. Kate Grabowski

**Affiliations:** 1 Department of Epidemiology, Johns Hopkins Bloomberg School of Public Health, Baltimore, Maryland, United States of America; 2 Rakai Health Sciences Program, Entebbe, Uganda; 3 Makerere University School of Public Health, Kampala, Uganda; 4 Department of Medicine, Division of Infectious Diseases, Johns Hopkins School of Medicine, Baltimore, Maryland, United States of America; 5 Department of Pathology, Johns Hopkins School of Medicine, Baltimore, Maryland, United States of America; 6 Division of Global HIV and TB, Centers for Disease Control and Prevention Uganda, Kampala, Uganda; 7 Department of Population and Family Health and Pediatrics, Columbia University, New York, New York, United States of America; 8 Laboratory of Immunoregulation, Division of Intramural Research, National Institute for Allergy and Infectious Diseases, National Institutes of Health, Bethesda, Maryland, United States of America; 9 Department of Epidemiology, UNC Gillings School of Global Public Health, Chapel Hill, North Carolina, United States of America; 10 Carolina Population Center, Chapel Hill, North Carolina, United States of America; National University of Singapore, SINGAPORE

## Abstract

Certain occupations have been associated with heightened risk of HIV acquisition and spread in sub-Saharan Africa, including female bar and restaurant work and male transportation work. However, data on changes in population prevalence of HIV infection and HIV incidence within occupations following mass scale-up of African HIV treatment and prevention programs is very limited. We evaluated prospective data collected between 1999 and 2016 from the Rakai Community Cohort Study, a longitudinal population-based study of 15- to 49-year-old persons in Uganda. Adjusted prevalence risk ratios for overall, treated, and untreated, prevalent HIV infection, and incidence rate ratios for HIV incidence with 95% confidence intervals were estimated using Poisson regression to assess changes in HIV outcomes by occupation. Analyses were stratified by gender. There were 33,866 participants, including 19,113 (56%) women. Overall, HIV seroprevalence declined in most occupational subgroups among men, but increased or remained mostly stable among women. In contrast, prevalence of untreated HIV substantially declined between 1999 and 2016 in most occupations, irrespective of gender, including by 70% among men (12.3 to 4.2%; adjPRR = 0.30; 95%CI:0.23–0.41) and by 78% among women (14.7 to 4.0%; adjPRR = 0.22; 95%CI:0.18–0.27) working in agriculture, the most common self-reported primary occupation. Exceptions included men working in transportation. HIV incidence similarly declined in most occupations, but there were no reductions in incidence among female bar and restaurant workers, women working in local crafts, or men working in transportation. In summary, untreated HIV infection and HIV incidence have declined within most occupational groups in Uganda. However, women working in bars/restaurants and local crafts and men working in transportation continue to have a relatively high burden of untreated HIV and HIV incidence, and as such, should be considered priority populations for HIV programming.

## Introduction

The scale-up of combination HIV treatment and prevention interventions (CHI) in sub-Saharan Africa has led to significant declines in HIV incidence [1–4]. However, rates of new HIV infection remain significantly above elimination thresholds in most countries [5,6]. Demographic heterogeneities in population-level risk of HIV acquisition and onward transmission likely drive continued virus spread, but they remain poorly characterized. A detailed understanding of such heterogeneities may facilitate targeted control efforts leading to further declines in HIV incidence and, ultimately, disease elimination.

Decades-old data established a person’s occupation as a salient risk factor for HIV acquisition in Africa. Occupations historically associated with increased HIV risk have included mining, bar work, truck driving, sex work, fishing, trading, and construction [3,4,7–10]. For example, a study of HIV risk in Uganda, conducted in 1992, prior to the availability of antiretroviral therapy (ART), found that bar and restaurant work, trading, and truck and taxi driving were associated with three times higher odds of HIV acquisition compared to agricultural work [4]. In southern Africa, truck driving, factory work, and mining have been strongly linked to higher HIV burden [10–12]. While historical studies have provided useful insights into HIV risk by occupation, there are very limited data comparatively assessing key HIV outcomes within occupational subgroups since the widespread rollout of HIV interventions in sub-Saharan Africa. Given that an individual’s occupation can be readily assessed in programmatic settings, understanding whether HIV burden currently varies by occupation may facilitate efficient targeting of interventions.

Here, we assessed the extent to which occupation-specific population prevalence of HIV and HIV incidence have changed since the implementation of combination HIV interventions (CHIs) including ART, using data from the Rakai Community Cohort Study (RCCS), a population-based HIV surveillance cohort in southern Uganda. We have previously measured trends in HIV prevalence and incidence in the RCCS and shown a 42% reduction in HIV incidence with ART rollout beginning in 2004 and VMMC scale-up beginning in 2007.13 However, it remains unclear whether or not untreated HIV prevalence and incidence declines have occurred uniformly across occupational subgroups in this population. We hypothesized that while the burdens of HIV, untreated HIV, and HIV incidence have declined within all occupations, heterogeneities in HIV outcomes by occupation persist.

## Methods

### Study population and procedures

The Rakai Community Cohort Study (RCCS) is conducted by the Rakai Health Sciences Program and is an open, population-based census and cohort study including consenting individuals aged 15–49 years across 40 communities in southern Uganda [13]. Individuals are followed at ~18-month intervals. Briefly, the RCCS conducts a household census to enumerate all individuals who are residents in the household, irrespective of presence or absence in the home at time of census, based on sex, age, and how long they have been resident in the community. The census is followed by a survey of residents aged 15 to 49 years. All RCCS participants provide written informed consent prior to interviews. Participant interviews provide self-reported data on socio-demographic characteristics, sexual behaviors, male circumcision status, and ART use. Two attempts are made to contact individuals who are censused and eligible but who do not participate in the surveys.

To determine individual participant HIV serostatus in RCCS, venous blood samples are obtained for HIV testing. Prior to October 2011, HIV testing used enzyme immunoassays (EIAs) with confirmation via western blot. Subsequently, a field-validated, parallel three-test, rapid HIV testing algorithm was introduced with demonstrated high sensitivity (>99.5%) and specificity (>99.5%). All rapid test positives in RCCS are confirmed by two EIAs, with western blot or PCR for discordant EIA results [14,15].

In this study, we included data from 12 consecutive RCCS survey rounds conducted between April 6, 1999, and September 2, 2016, collected from 30 continuously surveyed communities. The 12 surveys are herein denoted as Surveys 1 through 12: start and completion dates for each survey are included in S1 Table. Participation rates among census-eligible persons present in the community at the time of survey ranged from 74% to 98% (59%-66%, including those absent from the community) across survey rounds [16]. There were generally lower levels of participation in earlier survey rounds due to higher refusal rates. During the study period, participant retention (i.e., follow-up between consecutive survey rounds) decreased from 73% to 55% [16,17]. Loss to follow-up was due mostly to out-migration to non-eligible study communities. When considering only participants who were resident in the community at time of survey (e.g. excluding non-eligible migrants), retention decreased over the analysis from 93% to 80%.

For this study, RCCS data were accessed from December 15, 2018 through December 15, 2022. This study was approved by the Research and Ethics Committee of the Uganda Virus Research Institute and the Johns Hopkins School of Medicine Institutional Review Board. This study was also approved for the inclusion of children as ’research not involving greater than minimal risk’ with the permission of at least one parent.

### Measurement and classification of participant occupation

Occupational data were collected as self-reported primary occupations at the time of RCCS interviews. Participants were asked, “*What kind of work do you do*, *or what kind of activities keep you busy during an average day*, *whether you get money for them or not*.*”* There were 23 occupational subgroups that participants could select from on the questionnaire, including “*other*.” Individuals who listed “other” were asked to provide occupational details as a free-text response. Free-text responses were reviewed and re-assigned into pre-existing categories, or new categories were created as needed. There were 36 self-reported primary occupations, which were subsequently aggregated into 15 primary occupational subgroups (S2 Table). Of these larger subgroups, eight among men (agriculture, trading, student, construction, civil service, causal labor, mechanic, transportation) and nine among women (agriculture, trading, student, bar/restaurant work, civil service, hairdressing, local crafts, tailoring/laundry, housekeeping) contained a median number of ≥ 50 observations per survey across all surveys (S3A and S3B Table). These eight occupational subgroups among men and nine among women were the primary exposure units for all subsequent occupational analyses.

### Primary and secondary outcomes

Our primary study outcomes were (1) prevalent HIV infection, (2) prevalent untreated HIV infection, and (3) incident HIV infection. We defined prevalent HIV infection as any HIV infection in an individual (whether treated or untreated) and untreated HIV as HIV infection in an individual with HIV who did not self-report ART use at time of survey. We have previously shown that self-reported ART use has high specificity (99%) and moderate sensitivity (77%) in this population, and that this does not substantially vary by self-reported occupation [18]. We note that the prevalence of untreated HIV infection in the overall population (including seronegative individuals and persons living with HIV) was measured as a surrogate measure for population prevalence of viremia, which previous studies have shown is predictive of HIV incidence [16,19]. Incident HIV infection was defined as a first HIV seropositive test result in a person with a prior seronegative test result irrespective of HIV treatment status at first positive visit. The unit of analysis for HIV incidence was person-years of follow-up between surveys among persons who were initially HIV-seronegative and who contributed two consecutive survey visits or two visits with no more than one missing intervening survey. Incident infections were assumed to have occurred at the mid-point of the visit interval. Our secondary outcome was self-reported ART use among persons with HIV.

### Scale-up and measurement of combination HIV intervention coverage in Rakai

During the analysis period, ART rollout in Uganda, including Rakai, was phased as follows: in 2004, ART was offered to persons with a CD4-T-cell count of <250 cells/mm^3^; in 2011, the CD4 T-cell criterion was raised to <350; and in 2013, it was further increased to <500 and ART was also offered to all individuals with HIV, regardless of CD4 T-cell count, if they were pregnant, in a serodiscordant relationship, or self-identified as a sex worker or fisherfolk. The prevalence of self-reported ART use had risen to 69% among all persons with HIV by 2016. In addition to ART, the Rakai Health Sciences Program has provided free VMMC since 2007 to adolescents and men aged 13 years or older [16]. The prevalence of male circumcision increased from 15% in 1999 to 59% by 2016 [16]. Impacts of universal HIV test and treat and pre-exposure prophylaxis were not assessed in this study as implementation of these programs occurred after the analysis period in 2017 and 2018, respectively.

To assess changes in HIV incidence by occupation over calendar time, we divided the study period into pre-CHI (surveys 1–5; 1999–2004), early-CHI scale up (surveys 6–9; 2005–2011), and mature-CHI (surveys 10–12; 2011–2016) periods. Period-specific baselines were established as the first survey during each period, while the study baseline for individual participants was defined as their first survey during the entire study period.

### Statistical analysis

Demographic characteristics of participants at period-specific baselines were summarized using descriptive statistics, including median and interquartile ranges for continuous variables and frequencies and percentages for categorical variables. The prevalence of each primary occupation was estimated as the number of participants self-reporting that occupation, expressed as a proportion of all participants surveyed, and was stratified by sex. Self-reported ART use among participants with HIV was assessed during the early and mature-CHI periods and at the final study visit. Overall and untreated HIV prevalence were assessed at each of the 12 study visits and HIV incidence was estimated during the eleven inter-survey intervals over the 17-year analysis period. To evaluate changes in prevalence of untreated HIV infection and HIV incidence within occupational subgroups, we constructed log-binomial regression models to estimate prevalence risk ratios (PRR) and Poisson regression models to estimate incidence rate ratios (IRR). Because our primary objective was to describe patterns of HIV infection within occupational subgroups as opposed to causal inference, PRRs and IRRs were only adjusted for age and marital status to ensure demographic comparability across populations. We calculated IRRs for HIV infection, comparing incidence rates during the pre-, early-, and late-CHI periods. All statistical analyses were performed in Stata version 15 and the R statistical software (Version 3.6).

## Results

### Characteristics of study participants

Overall, 33,866 individuals (including 19,113 (56%) women) participated, contributing to a total of 102,759 person visits. Of these participants, 17,840 women and 14,244 men who were HIV-seronegative at their first study visit contributed 57,912 and 49,403 person-years to the incidence cohort, respectively. S4 Table shows characteristics of the study population by sex at the first (baseline) study visit within the CHI periods. Among women, during the pre-CHI baseline visit, median age was 25 years (IQR: 20–34), 59% (2056/3474) were married, and the prevalence of untreated HIV was 16%. Median age at the late-CHI baseline visit for women was somewhat older at 28 years (IQR: 22–34), 60% (2265/3758) were married, and prevalence of untreated HIV was 9.1%. Among men, during the first pre-CHI baseline visit, median age was 26 years (IQR: 20–33), 56% (1418/2518) were married, 15% (374/2518) were circumcised, and the prevalence of untreated HIV was 8.1%. In comparison, median age at the late-CHI baseline visit for men was 27 years (IQR: 20–36), 52% (1524/2944) were married, 46% (1359/2944) were circumcised, and the prevalence of untreated HIV was 6.4%.

### Population prevalence of occupations over calendar time

**Fig 1:**
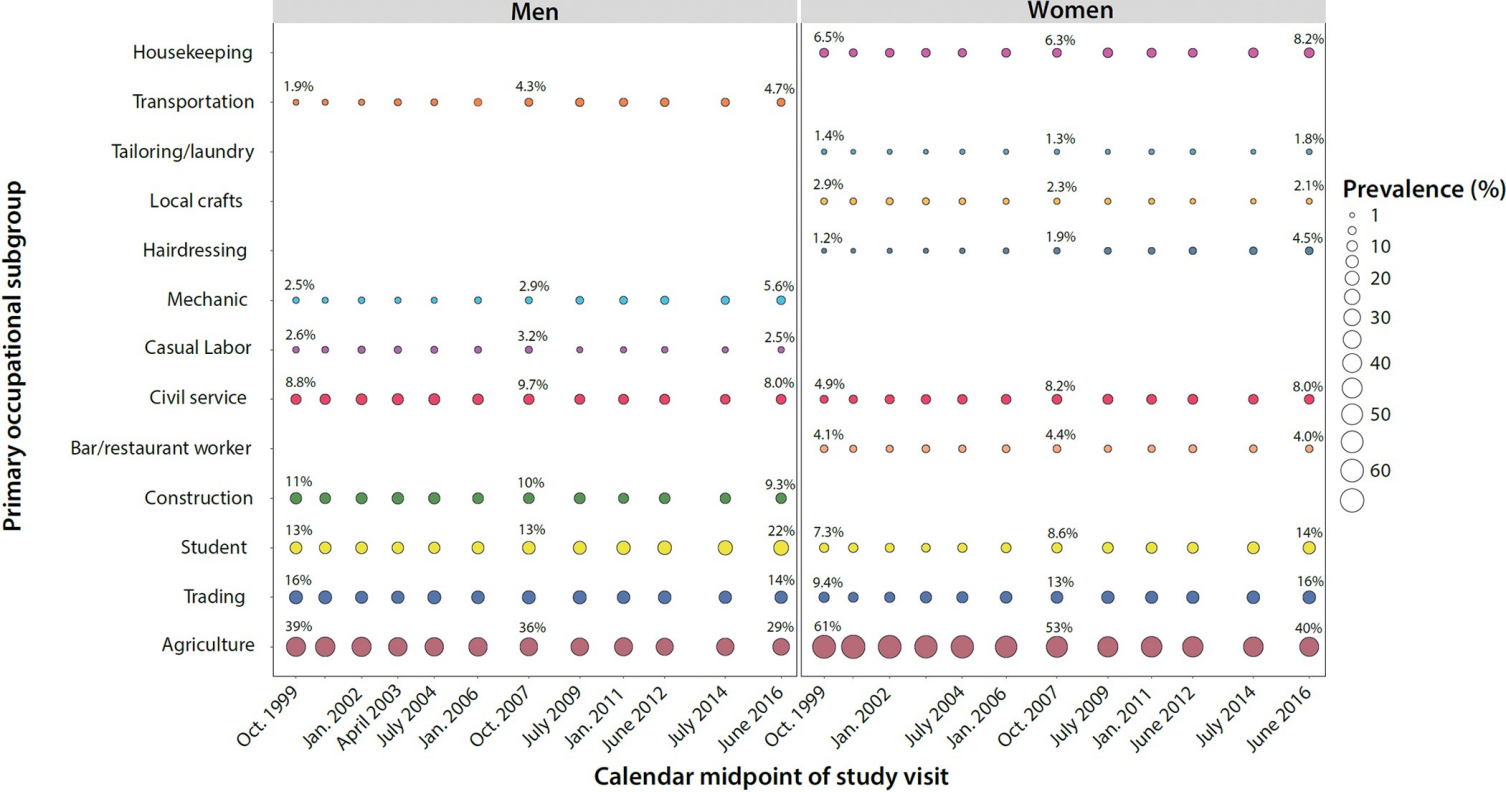
Prevalence of primary occupational subgroups by gender in the Rakai Community Cohort Study, 1999–2016.

Fig 1 shows the proportion of participants in each occupational subgroup over calendar time stratified by gender (see also S5A and S5B Table). At the initial visit (1999–2000), the majority of women (61%) reported agriculture as their primary occupation. While agriculture remained the most commonly reported female occupation at the final visit (2015–16), its prevalence significantly declined to 40% (PRR = 0.66; 95%CI: 0.62–0.69) (Fig 1). Declines in agricultural work among women were accompanied by an increase in the average age within the occupation (S1 Fig) and were predominately offset by the proportion of women who reported working in trading (9.4% in 1999 vs.16% in 2016, PRR = 1.7; 95%CI: 1.49–1.91) and being a student (7.3% vs. 14%, PRR = 1.97; 95%CI: 1.72–2.27). Notably, no women or men reported sex work as a primary occupation, and very few people reported being unemployed (n<7 at all study visits; S6A and S6B Table).

Men similarly reported agriculture and trading as their most common primary occupations (Fig 1). Between the first (1999–2000) and last (2015–2016) study visit, there was a decrease in the proportion of male participants reporting agriculture (39% vs. 29%, PRR = 0.74; 95%CI: 0.68–0.80), while a greater proportion reported being a student (13% vs. 22%, PRR = 1.74; 95%CI: 1.53–1.96), mechanic (2.5% vs. 5.6%, PRR = 2.29, 95%CI: 1.74–3.01), or working in transportation (1.9% vs. 4.7%, PRR = 2.42, 95%CI: 1.78–3.28).

### Trends in the prevalence of HIV, ART use, and untreated HIV within occupations

The prevalence of HIV remained unchanged in most occupational groups among women (Table 1), but increased among women working in agriculture (adjPRR = 1.19; 95%CI: 1.04–1.35) and decreased among hairdressers (adjPRR = 0.27; 95%CI: 0.18–0.41) and housekeepers (adjPRR = 0.68; 95%CI: 0.47–0.98). Among men, HIV prevalence decreased or trended downwards in most occupational groups but non-significantly trended upwards among men working in transportation (8.2% vs. 15.1%; adjPRR = 1.71; 95% CI: 0.64–4.58) and men working in casual labor (10.6% vs. 16.7%; adjPRR = 1.26; 95% CI: 0.58–2.73).

**Table 1:** Changes in prevalence of HIV infection between RCCS survey visit 1 (1999–2000) and RCCS survey visit 12 (2015–2016) by primary occupational subgroup and gender of study participants.

Occupational subgroup	Women N = 10,121					Men N = 7,876				
	Visit 1 (1999–2000), HIV prevalence, % (n/T) n = 3474	Visit 12 (2015–2016), HIV prevalence, % (n/T) n = 6647	Unadjusted PRR (95% CI)	adjPRR* (95% CI)	adjPRR p-value	Visit 1 (1999–2000), HIV prevalence, % (n/T) n = 2,518	Visit 12 (2015–2016), untreated HIV prevalence, % (n/T) n = 5,358	Unadjusted PRR (95% CI)	adjPRR** (95% CI)	adjPRR p-value
Agriculture	14.7 (313/2128)	18.0 (481/2669)	1.23 (1.08–1.40)	1.19 (1.04–1.35)	0.010	12.3 (120/975)	11.9 (183/1538)	0.97 (0.78–1.20)	0.83 (0.67–1.02)	0.081
Construction	-	-	-	-	-	12.6 (36/285)	10.8 (54/500)	0.86 (0.58–1.27)	0.64 (0.43–0.94	0.025
Trading	21.5 (70/325)	19.2 (201/1048)	0.89 (0.70–1.13)	0.82 (0.64–1.05)	0.112	12.7 (51/401)	11.0 (83/756)	0.86 (0.62–1.20)	0.59 (0.41–0.84)	0.004
Casual labor	-	-	-	-	-	10.6 (7/66)	16.7 (22/132)	1.57 (0.71–3.50)	1.26 (0.58–2.73)	0.558
Civil service	11.2 (19/170)	10.8 (57/529)	0.96 (0.59–1.57)	0.92 (0.54–1.58)	0.772	10.4 (23/221)	6.1 (26/429)	0.58 (0.34–1.00)	0.50 (0.30–0.83)	0.008
Student	2.4 (6/254)	3.1 (30/959)	1.32 (0.56–3.15)	0.91 (0.37–2.26)	0.837	0.6 (2/319)	0.5 (6/1178)	0.81 (0.17–4.01)	0.59 (0.12–2.99)	0.524
Mechanic	-	-	-	-	-	9.7 (6/62)	4.6 (14/302)	0.48 (0.19–1.20)	0.38 (0.15–0.99)	0.049
Transportation	-	-	-	-	-	8.2 (4/49)	15.1 (38/252)	1.85 (0.69–4.95)	1.71 (0.64–4.58)	0.286
Bar/Restaurant worker	34.7 (50/144)	41.6 (111/267)	1.20 (0.92–1.56)	1.22 (0.93–1.61)	0.158	-	-	-	-	-
Local crafts	19.8 (20/101)	24.8 (34/137)	1.25 (0.77–2.05)	0.98 (0.59–1.65)	0.945	-	-	-	-	-
Hairdressing	46.3 (19/41)	13.7 (41/300)	0.30 (0.19–0.46)	0.27 (0.18–0.41)	<0.001	-	-	-	-	-
Tailoring/laundry	4.0 (2/50)	13.1 (16/122)	3.28 (0.78–13.79)	2.49 (0.58–10.62)	0.218	-	-	-	-	-
Housekeeping	16.7 (38/227)	12.7 (69/545)	0.76 (0.53–1.09)	0.68 (0.47–0.98)	0.040	-	-	-	-	-
Other occupations	17.6 (6/34)	26.8 (19/71)	1.52 (0.66–3.46)	1.48 (0.63–3.50)	0.369	11.4 (16/140)	14.0 (38/271)	1.23 (0.71–2.12)	1.15 (0.66–1.99)	0.619
All occupations	15.6 (543/3474)	15.9 (1059/6647)	1.02 (0.93–1.12)	0.95 (0.86–1.04)	0.243	10.5 (265/2518)	8.7 (464/5358)	0.82 (0.71–0.95)	0.72 (0.62–0.83)	<0.001

PRR = prevalence risk ratios; adjPRR = adjusted prevalence risk; *Models adjusted for age and marital status of study participants.

The proportion of male and female participants with HIV self-reporting ART use increased over time among all occupational subgroups (Table 2A and 2B). During the late-CHI period and at the final study visit, levels of ART use were highest among women working in agriculture and lowest among female students. ART use was statistically significantly lower among female traders (adjPRR = 0.91; 95%CI: 0.83–0.98) and bar and restaurant workers (adjPRR = 0.87; 95%CI: 0.78–0.97) compared to women working in agriculture during the late CHI-period. Among men, ART use was highest among those working in civil service over the entire analysis period. During the late CHI period, ART use was statistically significantly lower among men working in trading (adjPRR = 0.91; 95%CI: 0.83–0.98) and male students (adjPRR = 0.59; 95%CI: 0.41–0.84) compared to men working in agriculture.

**Table 2:** a. Prevalence of self-reported ART use among women with HIV during the early and late-CHI periods and at the final study visit (Visit 12). b. Prevalence of self-reported ART use among men with HIV during the early and late-CHI periods and at the final study visit (Visit 12).

	Early–CHI (2004–2011) N = 3,352			Late–CHI (2011–2016) N = 2,695			Visit 12 N = 1,059		
	% self-reporting ART (n/T)	PRR (95% CI)	adjPRR (95% CI)	% self-reporting ART (n/T)	PRR (95% CI)	adjPRR (95% CI)	% self-reporting ART (n/T)	PRR (95% CI)	adjPRR (95% CI)
Agriculture	24.9 (432/1734)	Ref	Ref	64.6 (811/1256)	Ref	Ref	78.0 (375/481)	Ref	Ref
Trading	25.5 (149/584)	1.02 (0.87–1.20)	1.04 (0.89–1.22)	56.9 (302/531	0.88*** (0.81–0.96)	0.91** (0.83–0.98)	68.2 (137/201)	0.87** (0.79–0.97)	0.90** (0.81–0.99)
Casual labor	-	-	-	-	-	-	-	-	-
Civil service	19.5 (42/215)	0.78* (0.59–1.04)	0.89 (0.68–1.17)	58.2 (85/146)	0.90 (0.78–1.04)	0.93 (0.81–1.07)	70.2 (40/57)	0.90 (0.76–1.07)	0.93 (0.78–1.11)
Student	11.8 (2/17)	0.47 (0.13–1.74)	1.31 (0.35–4.92)	38.0 (19/50)	0.59*** (0.41–0.84)	0.83 (0.58–1.20)	53.3 (16/30)	0.68** (0.49–0.96)	0.86 (0.60–1.22)
Bar/restaurant worker	22.7 (65/287)	0.91 (0.72–1.14)	0.95 (0.76–1.20)	55.9 (160/286)	0.87** (0.78–0.97)	0.89** (0.80–0.99)	71.2 (79/111)	0.91 (0.80–1.04)	0.93 (0.82–1.06)
Local crafts	12.8 (12/94)	0.51** (0.30–0.88)	0.57** (0.34–0.95)	50.0 (32/64)	0.77** (0.60–0.99)	0.82 (0.65–1.05)	58.8 (20/34)	0.76* (0.57–1.00)	0.79 (0.60–1.05)
Hairdressing	21.0 (22/105)	0.84 (0.58–1.23)	1.13 (0.76–1.66)	54.3 (51/94)	0.84* (0.70–1.02)	0.95 (0.79–1.15)	65.9 (27/41)	0.85 (0.67–1.06)	0.92 (0.74–1.15)
Tailoring/laundry	23.7 (9/38)	0.95 (0.53–1.69)	1.07 (0.66–1.75)	60.0 (18/30)	0.93 (0.69–1.25)	0.99 (0.75–1.31)	75.0 (12/16)	0.96 (0.72–1.28)	1.01 (0.75–1.35)
Housekeeping	12.4 (28/226)	0.50*** (0.35–0.71)	0.73* (0.52–1.04)	52.5 (94/179)	0.81*** (0.70–0.94)	0.96 (0.83–1.11)	63.8 (44/69)	0.82** (0.68–0.98)	0.90 (0.75–1.08)
Other occupations	23.1 (12/52)	0.93 (0.56–1.53)	0.82 (0.50–1.33)	66.1 (39/59)	1.02 (0.85–1.24)	1.04 (0.85–1.27)	79.0 (15/19)	1.01 (0.80–1.28)	1.05 (0.82–1.36)
	**Early–CHI (2004–2011)** **N = 1,702**			**Late–CHI (2011–2016)** **N = 1,260**			**Visit 12** **N = 464**		
	**% self-reporting ART (n/T)**	**PRR** **(95% CI)**	**adjPRR** **(95% CI)**	**% self-reporting ART (n/T)**	**PRR** **(95% CI)**	**adjPRR** **(95% CI)**	**% self-reporting ART (n/T)**	**PRR** **(95% CI)**	**adjPRR** **(95% CI)**
Agriculture	21.2 (140/661)	Ref	Ref	54.4 (262/482)	Ref	Ref	64.5 (118/183)	Ref	Ref
Construction	7.8 (14/180)	0.37*** (0.22–0.62)	0.48*** (0.29–0.80)	41.0 (64/156)	0.76*** (0.62–0.93)	0.86 (0.70–1.06)	55.6 (30/54)	0.86 (0.66–1.12)	0.91 (0.69–1.19)
Trading	14.9 (47/316)	0.70** (0.52–0.95)	0.76* (0.57–1.01)	42.0 (94/224)	0.77*** (0.65–0.92)	0.80*** (0.67–0.94)	57.8 (48/83)	0.90 (0.73–1.11)	0.91 (0.74–1.12)
Casual labor	21.7 (15/69)	1.03 (0.64–1.64)	1.26 (0.79–2.02)	36.9 (24/65)	0.68** (0.49–0.94)	0.70** (0.51–0.96)	59.1 (13/22)	0.92 (0.64–1.32)	0.91 (0.64–1.29)
Civil service	24.5 (34/139)	1.16 (0.83–1.60)	0.96 (0.69–1.34)	60.6 (43/71)	1.11 (0.91–1.37)	1.03 (0.84–1.25)	84.6 (22/26)	1.31*** (1.08–1.60)	1.21* (1.00–1.48)
Student	33.3 (4/12)	1.57 (0.70–3.55)	11.83*** (4.72–29.68)	35.7 (5/14)	0.66 (0.32–1.33)	1.20 (0.56–2.55)	50.0 (3/6)	0.78 (0.35–1.74)	1.21 (0.49–2.96)
Mechanic	18.6 (11/59)	0.88 (0.51–1.53)	0.87 (0.54–1.40)	43.3 (13/30)	0.80 (0.53–1.21)	0.80 (0.52–1.23)	50.0 (7/14)	0.78 (0.45–1.32)	0.82 (0.50–1.34)
Transportation	15.4 (18/117)	0.73 (0.46–1.14)	1.01 (0.67–1.52)	42.9 (48/112)	0.79** (0.63–0.99)	0.94 (0.76–1.17)	52.6 (20/38)	0.82 (0.59–1.13)	0.94 (0.70–1.28)
Other occupations	16.1 (24/149)	0.76 (0.51–1.13)	0.83 (0.57–1.21)	50.9 (54/106)	0.94 (0.76–1.15)	1.01 (0.83–1.24)	60.5 (23/38)	0.94 (0.71–1.24)	0.99 (0.76–1.29)

**Fig 2:**
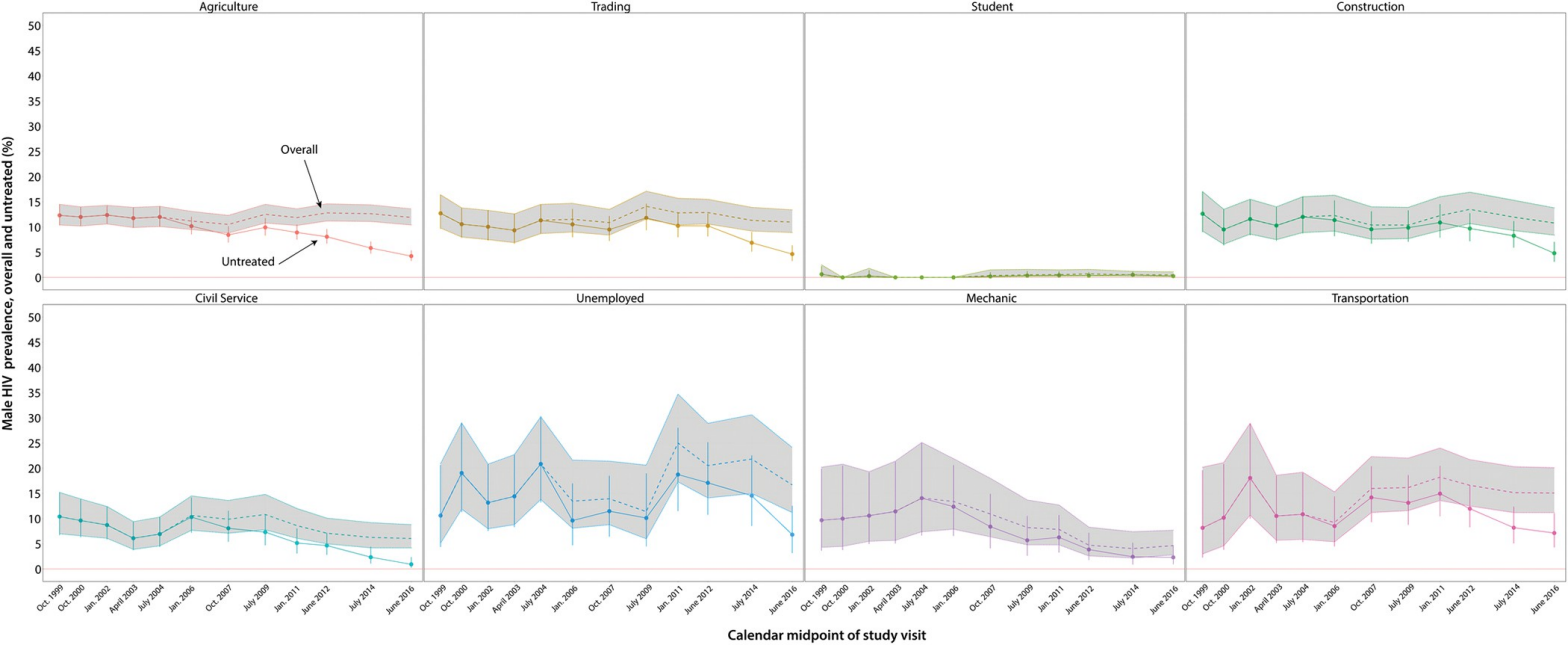
Trends in HIV prevalence (overall and untreated) among men by primary occupational subgroup in the Rakai Community Cohort Study (RCCS), 1999–2016; Untreated prevalence and 95% confidence intervals are shown as solid lines; overall HIV prevalence is shown as dashed lines with 95% confidence bands in gray. Data are plotted at the calendar midpoint of each study visit.

**Fig 3:**
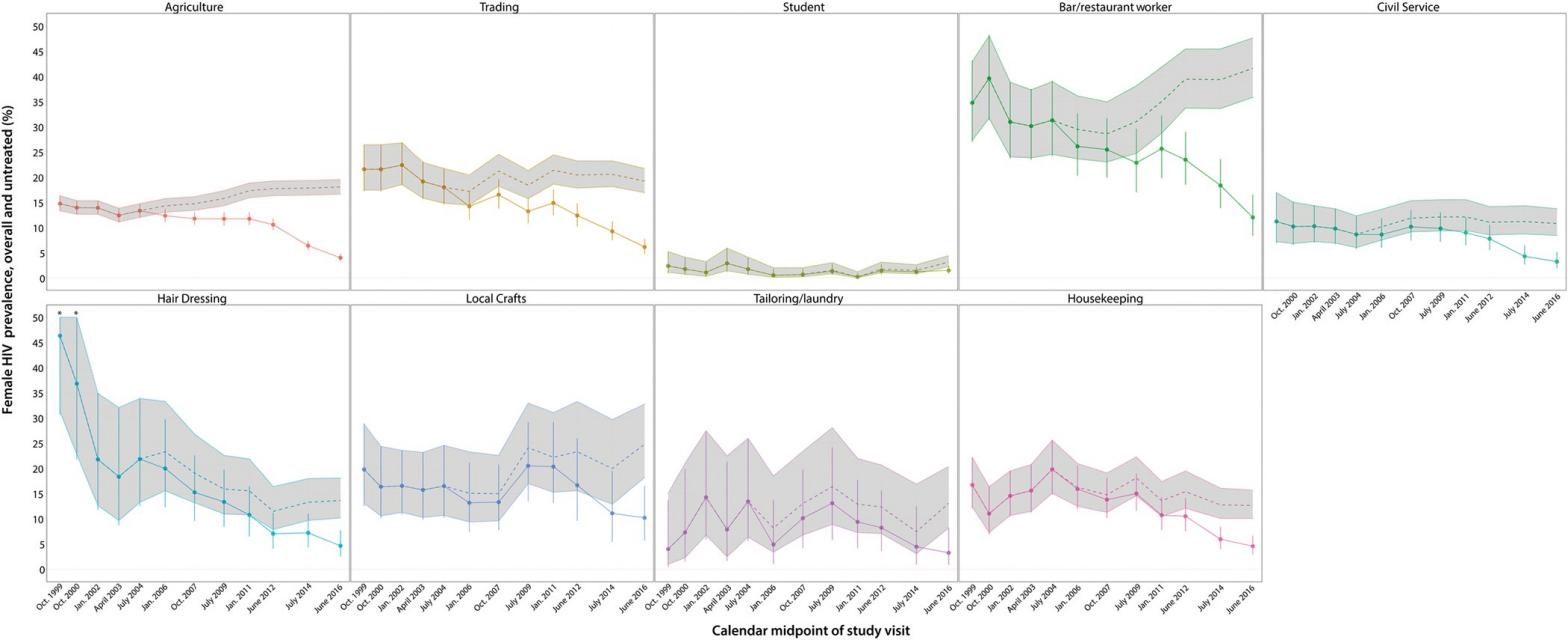
Trends in HIV prevalence (overall and untreated) among women by primary occupational subgroup in the Rakai Community Cohort Study (RCCS), 1999–2016; Untreated prevalence and 95% confidence intervals are shown as solid lines; overall HIV prevalence is shown as dashed lines with 95% confidence bands in gray. Data are plotted at the calendar midpoint of each study visit.

Figs 2 and 3 show the prevalence of untreated HIV within occupational subgroups among men and women at each of the 12 survey visits, respectively. Significant declines in the prevalence of untreated HIV were observed in nearly all occupational subgroups, irrespective of gender, with scale-up of ART use. Relative changes in untreated HIV prevalence between the first and final study visits are shown in Table 3 for each occupational subgroup by gender. The prevalence of untreated HIV significantly decreased within most occupations. For example, among women working in agriculture, prevalence of untreated HIV decreased from 14.7% to 4.0% (adjPRR = 0.22; 95%CI: 0.18–0.27), and among men, prevalence of untreated HIV decreased from 12.3% to 4.2% (adjPRR = 0.30, 95%CI: 0.23–0.41). Women working in bars and restaurants had among the highest HIV burdens across all occupational subgroups (Fig 3). The prevalence of untreated HIV significantly declined among female bar and restaurant workers from a high of 34.7% in 1999–2000 to 12.0% by 2015–2016 (adjPRR = 0.38; 95%CI: 0.25–0.58) (Table 3). However, these women had a 41.6% overall HIV seroprevalence at the final study visit in 2016 and still maintained a three-fold higher burden of untreated HIV compared to women working in agriculture at the final versus initial visits (12.0% versus 4.0%). Women working in local crafts and in trading also continued to have a high prevalence of untreated HIV compared to women in agriculture at the final visit (Table 3). Men working in transportation did not have significantly higher HIV prevalence than other male occupations at the initial visit (Table 3). However, we observed no declines in untreated HIV in this population over the analysis period, and by the final visit, they had the highest prevalence of untreated HIV among all male occupations at 7.1%.

**Table 3:** Changes in prevalence of untreated HIV infection between RCCS survey visit 1 (1999–2000) and RCCS survey visit 12 (2015–2016) by primary occupational subgroup and gender of study participants.

Occupational subgroup	Women N = 10,121					Men N = 7,876				
	Visit 1 (1999–2000), untreated HIV prevalence, % (n/T) n = 3474	Visit 12 (2015–2016), untreated HIV prevalence, % (n/T) n = 6647	Unadjusted PRR (95% CI)	adjPRR* (95% CI)	adjPRR p-value	Visit 1 (1999–2000), untreated HIV prevalence, % (n/T) n = 2,518	Visit 12 (2015–2016), untreated HIV prevalence, % (n/T) n = 5,358	Unadjusted PRR (95% CI)	adjPRR** (95% CI)	adjPRR p-value
Agriculture	14.7 (313/2128)	4.0 (106/2669)	**0.27 (0.22–0.33)**	**0.22 (0.18–0.27)**	**<0.001**	12.3 (120/975)	4.2 (65/1538)	**0.34 (0.26–0.46)**	**0.30 (0.23–0.41)**	**<0.001**
Construction	-	-	-	-	-	12.6 (36/285)	4.8 (24/500)	**0.38 (0.23–0.62**	**0.27 (0.17–0.43**	**<0.001**
Trading	21.5 (70/325)	6.1 (64/1048)	**0.28 (0.21–0.39)**	**0.09 (0.06–0.12)**	**<0.001**	12.7 (51/401)	4.6 (35/756)	**0.36 (0.24–0.55)**	**0.27 (0.17–0.43)**	**<0.001**
Casual labor	-	-	-	-	-	10.6 (7/66)	6.8 (9/132)	0.64 (0.25–1.65)	0.54 (0.22–1.35)	0.185
Civil service	11.2 (19/170)	3.2 (17/529)	**0.29 (0.15–0.54)**	**0.07 (0.04–0.15)**	**<0.001**	10.4 (23/221)	0.9 (4/429)	**0.09 (0.03–0.26)**	**0.09 (0.03–0.25)**	**<0.001**
Student	2.4 (6/254)	1.5 (14/959)	**0.62 (0.24–1.59)**	**0.11 (0.04–0.30)**	**<0.001**	0.6 (2/319)	0.3 (3/1178)	0.41 (0.07–2.42)	0.23 (0.04–1.19)	0.079
Mechanic	-	-	-	-	-	9.7 (6/62)	2.3 (7/302)	**0.24 (0.08–0.69)**	**0.22 (0.07–0.67)**	**0.007**
Transportation	-	-	-	-	-	8.2 (4/49)	7.1 (18/252)	0.88 (0.31–2.47)	0.90 (0.30–2.73)	0.858
Bar/Restaurant worker	34.7 (50/144)	12.0 (32/267)	**0.35 (0.23–0.51)**	**0.21 (0.14–0.31)**	**<0.001**	-	-	-	-	-
Local crafts	19.8 (20/101)	10.2 (14/137)	**0.52 (0.27–0.97)**	**0.29 (0.14–0.58)**	**<0.001**	-	-	-	-	-
Hairdressing	46.3 (19/41)	4.7 (14/300)	**0.10 (0.05–0.19)**	**0.01 (0.01–0.02)**	**<0.001**	-	-	-	-	-
Tailoring/laundry	4.0 (2/50)	3.3 (4/122)	0.82 (0.16–4.33)	0.25 (0.05–1.35)	0.107	-	-	-	-	-
Housekeeping	16.7 (38/227)	4.6 (25/545)	**0.27 (0.17–0.44)**	**0.11 (0.07–0.17)**	**<0.001**	-	-	-	-	-
Other occupations	17.6 (6/34)	5.6 (4/71)	0.32 (0.10–1.06)	0.28 (0.08–1.02)	0.053	11.4 (16/140)	5.5 (15/271)	**0.48 (0.25–0.95)**	**0.48 (0.25–0.96)**	**0.037**
All occupations	15.6 (543/3474)	4.4 (294/6647)	**0.28 (0.25–0.32)**	**0.27 (0.24–0.31)**	**<0.001**	10.5 (265/2518)	3.4 (180/5358)	**0.32 (0.27–0.38)**	**0.29 (0.24–0.35)**	**<0.001**

PRR = prevalence risk ratios; adjPRR = adjusted prevalence risk; *Models adjusted for age and marital status of study participants.

### Changes in HIV incidence within occupations before and during scale-up of CHI programs

Table 4 shows HIV incidence by occupation, gender, and calendar time. In the early CHI period, HIV incidence rates ranged from 0.4 to 2.3 per 100 person-years between occupational subgroups among women, and from 0.1 to 1.8 per 100 person-years among men. Between the early and late CHI periods, HIV incidence declined or trended downwards among most occupational subgroups. For example, among those working in agriculture, HIV incidence declined by 67% among men (adjIRR = 0.33; 95%CI: 0.21–0.54) and 38% among women (adjIRR = 0.62; 95%CI: 0.45–0.86). HIV incidence trends in most other occupations showed a decline, but were not statistically significant. While HIV incidence did not decline among students, incidence in this population was low overall. HIV incidence rates also did not decline among men working in transportation, and women working in bars and restaurants or local crafts. S7 Table shows the adjusted relative risk of HIV acquisition by occupation during the late CHI period. Compared to women working in agriculture, female bar and restaurant workers had a three-fold higher rate of HIV incidence (adjIRR = 2.88; 95%CI: 1.51–5.49). Men working in transportation also had significantly higher HIV incidence compared to agricultural workers (adjIRR = 2.75; 95% CI: 1.37–5.50). Regardless of sex, students had a significantly lower risk of HIV acquisition compared to persons working in agriculture (men: adjIRR = 0.19; 95% CI: 0.05–0.73; women: adjIRR = 0.36; 95% CI: 0.18–0.72).

**Table 4:** Incidence of HIV infection by primary occupational subgroup, sex, and CHI (combination HIV intervention) calendar period.

	**Women (N = 17,840)**						
**Occupation**	**Incidence rate per 100 py (n/py)**			**IRR (95% CI)**		**adjIRR (95%CI)**	
	**Pre–CHI** **(1999–2004)**	**Early–CHI** **(2004–2011)**	**Late–CHI** **(2011–2016)**	**Early–CHI vs. Pre-CHI (ref)**	**Late–CHI vs. Pre-CHI (ref)**	**Early–CHI vs. Pre-CHI (ref)**	**Late–CHI vs. Pre-CHI (ref)**
Agriculture	1.1 (97/8490)	0.9 (129/13785)	0.7 (62/9515)	0.82 (0.63–1.07)	0.57^*^ **(0.41–0.79)**	0.87 (0.67–1.13)	0.62^*^ **(0.45–0.86)**
Bar/restaurant worker	1.1 (4/365)	2.1 (17/813)	2.0 (12/605)	1.91 (0.64–5.69)	1.81 (0.58–5.66)	2.13 (0.72–6.36)	2.79 (0.85–9.19)
Trading	1.4 (17/1222)	1.4 (49/3474)	0.7 (23/3117)	1.01 (0.58–1.77)	0.53^*^ **(0.28–1.00)**	1.19 (0.69–2.06)	0.72 (0.38–1.37)
Hairdressing	2.3 (3/133)	1.6 (10/624)	0.8 (6/774)	0.71 (0.19–2.62)	0.34 (0.08–1.39)	0.73 (0.20–2.68)	0.36 (0.09–1.43)
Civil service	1.1 (9/851)	0.6 (14/2399)	0.3 (5/1849)	0.55 (0.24–1.28)	0.26^*^ **(0.09–0.77)**	0.76 (0.31–1.86)	0.49 (0.13–1.78)
Student	0.3 (2/623)	0.4 (6/1433)	0.7 (14/2059)	1.30 (0.26–6.48)	2.12 (0.48–9.35)	1.23 (0.25–6.11)	1.93 (0.44–8.36)
Housekeeping	1.2 (6/496)	1.1 (15/1388)	0.9 (12/1278)	0.89 (0.35–2.32)	0.78 (0.29–2.08)	1.0 (0.37–2.67)	0.89 (0.30–2.59)
Local crafts	1.3 (5/387)	2.3 (12/518)	1.6 (5/308)	1.79 (0.63–5.13)	1.25 (0.36–4.38)	1.88 (0.65–5.44)	1.36 (0.38–4.91)
Tailoring/laundry	1.7 (2/121)	1.4 (5/355)	1.1 (3/261)	0.86 (0.16–4.47)	0.70 (0.12–4.23)	0.94 (0.17–5.10)	0.85 (0.15–4.84)
Other occupations	1.1 (1/93)	0.0 (0/169)	1.2 (5/406)	-	1.14 (0.13–9.96)	-	1.41 (0.16–12.48)
All occupations	1.1 (146/12781)	1.0 (257/24958)	0.7 (147/20173)	0.90 (0.74–1.11)	0.64^*^ **(0.51–0.80)**	0.93 (0.76–1.14)	0.66^*^ **(0.53–0.83)**
	**Men (N = 14,244)**						
	**Incidence rate per 100 py (n/py)**			**IRR (95% CI)**		**adjIRR (95%CI)**	
	**Pre–CHI** **(1999–2004)**	**Early–CHI** **(2004–2011)**	**Late–CHI** **(2011–2016)**	**Early–CHI vs. Pre-CHI (ref)**	**Late–CHI vs Pre-CHI (ref)**	**Early–CHI vs. Pre-CHI (ref)**	**Late–CHI vs Pre-CHI (ref)**
Agriculture	1.4 (53/3894)	0.8 (62/8046)	0.4 (25/5834)	0.57^*^ **(0.39–0.82)**	0.32^*^ **(0.20–0.51)**	0.58^*^ **(0.40–0.84)**	0.33^*^ **(0.21–0.54)**
Construction	1.6 (18/1128)	1.2 (28/2322)	0.5 (9/1690)	0.76 (0.42–1.37)	0.33^*^ **(0.15–0.75)**	0.79 (0.42–1.47)	0.35^*^ **(0.15–0.83)**
Trading	0.8 (13/1541)	0.7 (26/3613)	0.5 (15/2746)	0.85 (0.44–1.67)	0.65 (0.31–1.37)	0.89 (0.46–1.70)	0.69 (0.33–1.45)
Casual labor	1.2 (3/259)	1.6 (7/431)	0.6 (2/322)	1.40 (0.36–5.49)	0.54 (0.09–3.25)	1.67 (0.42–6.66)	0.68 (0.11–4.30)
Civil service	0.7 (7/981)	0.6 (12/2121)	0.4 (6/1673)	0.79 (0.31–2.02)	0.50 (0.17–1.50)	0.78 (0.31–2.00)	0.49 (0.16–1.51)
Student	0.1 (1/955)	0.05 (1/1905)	0.1 (3/2840)	0.50 (0.03–8.03)	1.01 (0.11–9.71)	0.51 (0.03–8.23)	0.44 (0.04–4.86)
Mechanic	0.0 (0/228)	1.0 (8/780)	0.4 (4/901)	-	-	-	-
Transportation	1.4 (4/287)	1.8 (21/1181)	1.2 (12/964)	1.28 (0.43–3.75)	0.89 (0.29–2.80)	1.33 (0.45–3.91)	1.10 (0.35–3.50)
Other occupations	1.8 (10/546)	1.9 (21/1099)	0.9 (10/1115)	1.04 (0.49–2.23)	0.49 (0.20–1.19)	1.06 (0.50–2.26)	0.50 (0.21–1.23)
All occupations	1.1 (109/9821)	0.9 (186/21498)	0.5 (86/18085)	0.78^*^ **(0.62–0.99)**	0.43^*^ **(0.32–0.57)**	0.79^*^ **(0.62–1.0)**	0.44^*^ **(0.33–0.59)**

py = person years; IRR = incidence rate ratio; adjIRR = adjusted incidence rate ratio for age and marital status; IRR not presented for other occupations (women, Early-CHI) and mechanic (men) because there were no cases in the numerator and denominator respectively; CHI = combination HIV intervention

*p<0.05.

## Discussion

In this population-based study, overall prevalence of HIV (treated and untreated) mostly declined among men, but remained stable or increased in most occupational subgroups among women. We also observed declining prevalence of untreated HIV and HIV incidence among most occupational subgroups with the scale up of HIV treatment and prevention programs in Uganda. Among men and women working in agriculture, the most common self-reported primary occupation, prevalence of untreated HIV and HIV incidence declined by more than two-thirds. However, this downward trend was not always the case for other occupations. While women working in bars and restaurants made up a small proportion of the overall population, they had among the highest burdens of untreated HIV prior to HIV intervention scale-up, with no declines in HIV incidence over the analysis period. We also found no significant reduction in HIV incidence among male transportation workers. Moreover, both female bar and restaurant workers and male transportation workers had the highest prevalence of untreated HIV at the final study visit. HIV incidence rates among women reporting student and crafting as primary occupations also showed no decrease following CHI scale-up, although students had a very low HIV burden overall. Taken together, these results suggest that members of traditionally high-risk occupations continue to experience elevated rates of HIV incidence and remain sub-optimally served by HIV programs.

Other studies have reported high HIV prevalence among female bar workers in sub-Saharan Africa [20,21]. In this study, HIV prevalence among female bar and restaurant workers exceeded 40% with rising prevalence in recent years. While the prevalence of untreated HIV significantly declined in this population, it was three times higher than among women working in agriculture at the final study visit. The high burden of HIV among these women has been linked to female sex work, alcohol use, and mobility [22–24]. In a systematic review of socio-demographic characteristics and risk factors for HIV among female bar workers, high rural-to-urban mobility, transactional sex, and inconsistent condom use were common and associated with financial needs and social marginalization [22]. Our results underscore that female bar and restaurant workers should be a priority population for African HIV treatment and prevention programs. While key population-based programs in Africa include female sex workers, and female bar workers are often engaged in sex work, not all women working in bars and restaurants at high risk of HIV classify themselves as sex workers [22]. Multi-level, social influence, and structural HIV prevention interventions targeting alcohol-serving establishments, including enhanced sexually transmitted infection clinic services, portable health services, and peer education, have been shown to be effective in settings outside Africa, for reducing HIV risk and increasing treatment uptake [25,26].

Prior research has shown that men working in transportation are highly mobile and often engage in transactional sex [27–29]. We found that the prevalence of untreated HIV did not significantly decline in this occupational sub-group with the increasing availability of treatment and prevention. Prior research has linked male transportation workers, including truck drivers, to higher risk of HIV transmission [27], and has shown that men working in this occupation frequently engage with sex workers and women working in bars and restaurants [28,30]. Supplies of free condoms, roadside clinics, and free HIV testing services at truck stops are some HIV prevention interventions that have been targeted to male transportation workers [10,30]; however, levels of awareness and uptake of such services in this population have been low [10,31].

Adolescent girls and young women aged 15 to 24 years have a disproportionately high risk of HIV acquisition in Africa [32–35], but HIV risk was significantly lower among young people who list their occupation as “student” and who have higher education attainment, regardless of sex [36–39]. During the study period, HIV prevalence declined in female students by nearly 90%. Incidence of HIV remained stable for both male and female students, but compared to those in agriculture, students of both sexes had lower HIV incidence during the late-CHI period. Research from South Africa has shown that students tend to have smaller sexual networks and are less likely to report high-risk sexual behaviors compared to those not in school [37]. Lower HIV incidence and prevalence among female students have also been attributed to avoiding the consequences of unprotected sex and increased self-efficacy for negotiating safer sex with their partners [40]. Interventions that increase school enrollment of adolescent girls and young women may decrease sexual initiation, high-risk sexual behavior, and HIV risk [32].

Since the onset of the COVID-19 pandemic in Uganda during the spring of 2020, schools remained fully or partially closed until 2022. A review of adolescent sexual and reproductive health during the COVID-19 pandemic found an increase in teenage pregnancies and gender-based violence [41]. Given the strong protective effects of schooling on HIV acquisition, understanding the extent to which school closures impact HIV and other reproductive health outcomes, such as unplanned pregnancy, is an urgent public health priority.

Earlier studies have established migration and mobility as a key risk factor for HIV acquisition and transmission [23,42,43]. Overall, we found that the occupations which tend to have high mobility also had higher prevalence of untreated HIV and HIV incidence despite scale-up of HIV interventions. Both female bar and restaurant work and male transportation work are associated with increased mobility as well as high-risk sexual behaviors, including concurrent sexual partnerships and inconsistent condom use [28,29,44]. Specialized service-delivery tailored to mobile populations, such as client-managed groups, adherence clubs, community drug distribution points, and multi-month prescriptions may reduce HIV burden in these populations [45–47].

The shifting distribution of the occupational makeup in our study population away from agriculture likely reflects the increasing urbanization happening across the African continent [48]. Little data exists on the impact of urbanization on HIV transmission; however, in sub-Saharan Africa, HIV prevalence and incidence have been reported to be higher in urban than in rural centers [49,50]. This has been attributed to factors such as relative affluence in urban centers, increased social interaction, and higher-risk behaviors such as transactional sex and concurrent sexual partnerships [51–53]. More research is needed to elucidate the impact of increasing urbanization on HIV transmission within African populations.

Our study has important limitations. First, both occupation and ART use were self-reported and may be subject to bias. However, we have previously shown that self-reported ART use has high specificity and moderate sensitivity in this same study population, and does not substantially vary by self-reported occupation [18]. Second, female sex work in Uganda is criminalized and was likely substantially underreported in our survey [4]. Third, PEPFAR-supported key population HIV prevention programs began in this region in 2017, after the time of the analysis, and so their impact cannot be assessed. Given previously reported links between female sex work and bar work [21], our findings support PEPFAR’s ongoing focus on targeted HIV prevention and treatment to female sex workers. However, many bar workers do not identify as sex workers (none in this study), suggesting that they and other population sub-groups may merit additional programmatic consideration. Neither bar and restaurant workers nor male transportation workers are presently considered priority populations for HIV programming in Uganda. Fourth, while the longitudinal nature of this study is a strength, analysis of incident HIV infections were limited by a small number of events in some occupational subgroups, which may have obscured significant trends. Additionally, non-differential non-response and loss to follow-up may have biased our results but in earlier studies from this same population, sensitivity analyses showed little to no impact of selection bias on incidence estimates [16]. Lastly, because participants become aware of the risk of contracting HIV, their HIV status, and available treatments and prevention through their participation in the study, they may be more likely to take up and adhere to preventative measures or treatment, and so our results may not be generalizable to other populations. However, we expect the Hawthorne effect to be limited in this open cohort with substantial in- and out-migration.

In summary, prevalence of untreated HIV infection and HIV incidence declined in most occupational subgroups following the mass scale-up of HIV prevention and treatment interventions in rural southern Uganda. However, HIV burden remained relatively high in some occupations, including the traditionally high-risk occupations of female bar and restaurant work and male transportation work. HIV programs that meet the unique needs of these high-risk populations, which tend to be more mobile with higher levels of HIV-associated risk behaviors, may help achieve HIV epidemic control.

## Supporting information

S1 Checklist Inclusivity in global research. (DOCX)

S2 Checklist STROBE Statement—checklist of items that should be included in reports of observational studies. (DOCX)

S1 Fig Boxplots of age in years at each study visit, among RCCS agricultural workers. (TIF)

S1 Table Rakai Community Cohort Study (RCCS) survey start and end dates. (DOCX)

S2 Table Recategorization of 36 self-reported primary occupations into occupational sub-groups. (DOCX)

S3 Table A. Number of male observations at each study visit by primary occupational subgroup. B. Number of female observations in each primary occupational subgroup at each study visit.
(DOCX)

S4 Table Characteristics of the study population at the baseline visit within each CHI calendar period by gender. (DOCX)

S5 Table A. Prevalence (%) of major occupations among women by visit. B. Prevalence (%) of major occupations among men by visit.
(DOCX)

S6 Table A. Self-reported primary occupations by male RCCS study participants at each study visit. B. Self-reported primary occupations by female RCCS study participants at each study visit.
(DOCX)

S7 Table Adjusted incidence rate ratios of HIV infection comparing all occupations vs. agriculture during the late-CHI period. (DOCX)
