# Supplementary figures and images for: HIV epidemiologic trends among occupational groups in Rakai, Uganda: A population-based longitudinal study, 1999–2016
Source: PLOS Glob Public Health. 2024 Feb 20;4(2):e0002891. doi: 10.1371/journal.pgph.0002891 (PMC10878534; doi:10.1371/journal.pgph.0002891)

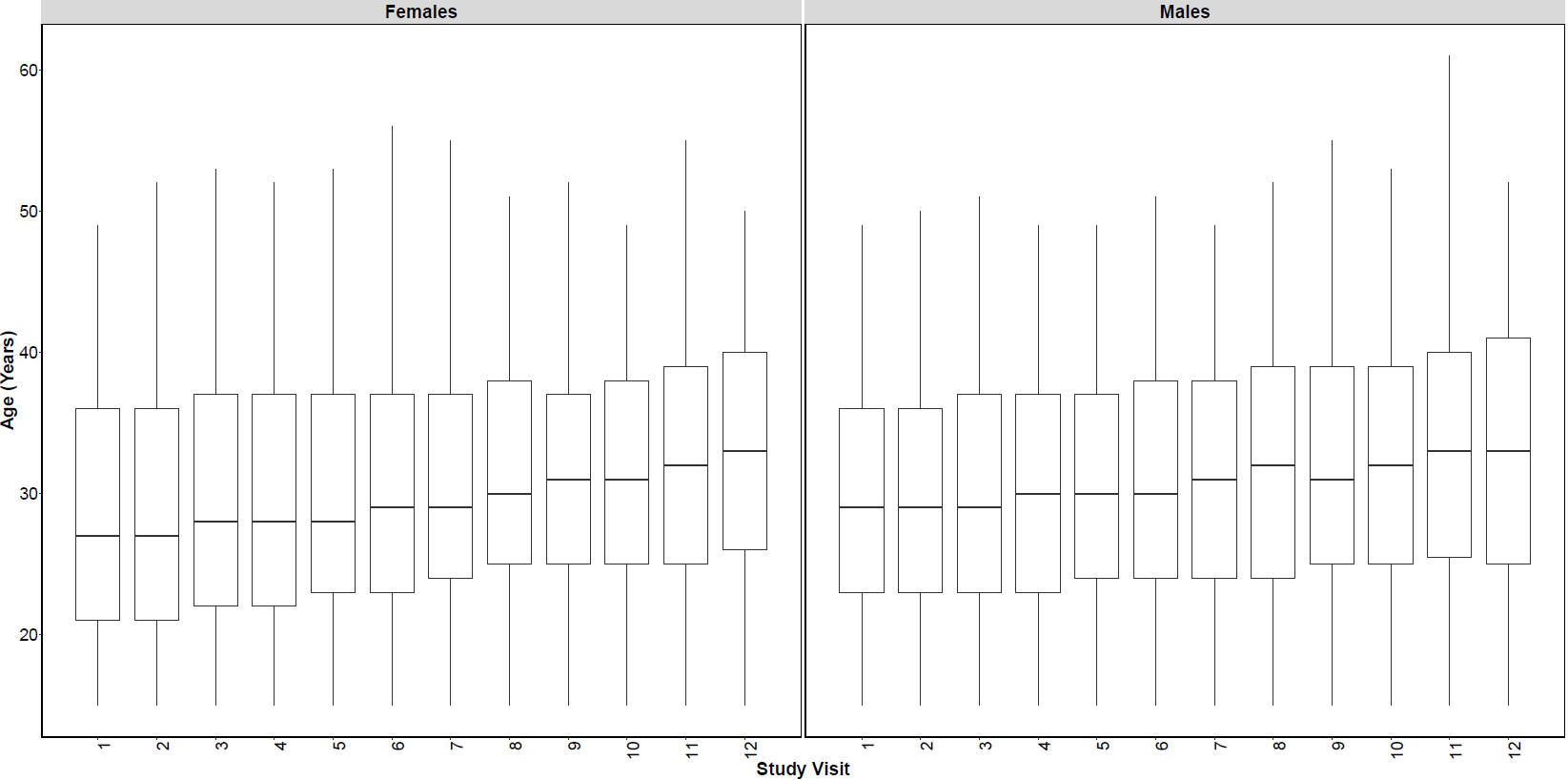

Supplement: S1 Fig — (TIF) [file pgph.0002891.s003.tif]
