# Supplementary material for: HIV epidemiologic trends among occupational groups in Rakai, Uganda: A population-based longitudinal study, 1999–2016
Source: PLOS Glob Public Health. 2024 Feb 20;4(2):e0002891. doi: 10.1371/journal.pgph.0002891 (PMC10878534; doi:10.1371/journal.pgph.0002891)
Supplement: S1 Table — (DOCX) [file pgph.0002891.s004.docx]

**HIV epidemiologic trends among occupational groups in Rakai, Uganda: A population-based longitudinal study, 1999-2016**

Victor O. Popoola^1^, Joseph Kagaayi^2,3^, Joseph Ssekasanvu^1,2^, Robert Ssekubugu^2^, Grace Kigozi^2^, Anthony Ndyanabo^2^, Fred Nalugoda^2^, Larry W. Chang^1,2,4^, Tom Lutalo^2^, Aaron A.R. Tobian^1,5^, Donna Kabatesi^6^, Stella Alamo^6^, Lisa A. Mills^6^, Godfrey Kigozi^2^, Maria J. Wawer^1,2^, John Santelli^7^, Ronald H. Gray^1,2^, Steven J Reynolds^4,8^, David Serwadda^2,3^, Justin Lessler^1,9,10^, M. K. Grabowski^1,2,5^

1. Department of Epidemiology, Johns Hopkins Bloomberg School of Public Health, Baltimore, Maryland
2. Rakai Health Sciences Program, Entebbe, Uganda
3. Makerere University School of Public Health, Kampala, Uganda
4. Division of Infectious Diseases, Department of Medicine, Johns Hopkins School of Medicine, Baltimore, Maryland, United States of America
5. Department of Pathology, Johns Hopkins School of Medicine, Baltimore, Maryland, United States of America
6. Division of Global HIV and TB, Centers for Disease Control and Prevention Uganda, Kampala Uganda
7. Department of Population and Family Health and Pediatrics, Columbia University, New York, New York, United States of America
8. Laboratory of Immunoregulation, Division of Intramural Research, National Institute for Allergy and Infectious Diseases, National Institutes of Health, Bethesda, Maryland, United States of America
9. Department of Epidemiology, UNC Gillings School of Global Public Health, Chapel Hill, North Carolina, United States of America
10. Carolina Population Center, Chapel Hill, North Carolina, United States of America

**S1 Table. Rakai Community Cohort Study (RCCS) survey start and end dates.**

| **RCCS survey** | **Start Date** | **End Date** |
| --- | --- | --- |
| **Pre-CHI** | | |
| 1 | 04-06-1999 | 02-14-2000 |
| 2 | 02-15-2000 | 05-26-2001 |
| 3 | 04-03-2001 | 05-31-2002 |
| 4 | 07-15-2002 | 08-01-2003 |
| 5 | 09-26-2003 | 11-23-2004 |
| **Early-CHI** | | |
| 6 | 02-15-2005 | 06-30-2006 |
| 7 | 08-30-2006 | 06-06-2008 |
| 8 | 06-06-2008 | 12-07-2009 |
| 9 | 01-18-2010 | 06-24-2011 |
| **Late-CHI** | | |
| 10 | 08-10-2011 | 10-22-2013 |
| 11 | 04-18-2013 | 01-30-2015 |
| 12 | 01-25-2015 | 09-02-2016 |
| CHI=combination HIV intervention | | |
